# Formal Comment on “*Mitigation of endemic GI-tract pathogen-mediated inflammation through development of multimodal treatment regimen and its impact on SIV acquisition in rhesus macaques*” by Bochart et al. (2021)

**DOI:** 10.1371/journal.ppat.1010831

**Published:** 2022-09-27

**Authors:** Rudolf P. Bohm, Matthew W. Breed, Joyce K. Cohen, Andrew J. Haertel, Lisa C. Halliday, Joshua A. Kramer, Mia T. Lieberman, Kelly A. Rice, Jeffery A. Roberts, Kasi E. Russell-Logrigue, Gregory W. Salyards, Diana G. Scorpio, J. Scott Weese

**Affiliations:** 1 Tulane National Primate Research Center, Tulane University, Covington, Louisiana, United States of America; 2 Laboratory Animal Sciences Program, Frederick National Laboratory for Cancer Research, Bethesda, Maryland, United States of America; 3 The Emory National Primate Research Center, Emory University, Atlanta, Georgia, United States of America; 4 Oregon National Primate Research Center, Oregon Health & Science University, Beaverton, Oregon, United States of America; 5 Biologic Resources Laboratory, University of Illinois at Chicago, Chicago, Illinois, United States of America; 6 Harvard Center for Comparative Medicine, Harvard Medical School, Boston, Massachusetts, United States of America; 7 Center for Biologics Evaluation and Research, Food and Drug Administration, Silver Spring, Maryland, United States of America; 8 California National Primate Research Center, University of California Davis, Davis, California, United States of America; 9 Division of Veterinary Resources, National Institutes of Health, Bethesda, Maryland, United States of America; 10 Southwest National Primate Research Center, Texas Biomedical Research Institute, San Antonio, Texas, United States of America; 11 Ontario Veterinary College, University of Guelph, Guelph, Canada; University of Minnesota, UNITED STATES

## Introduction

In this letter, we raise concerns regarding the recent publication, “*Mitigation of endemic GI-tract pathogen-mediated inflammation through development of multimodal treatment regimen and its impact on SIV acquisition in rhesus macaques*” [1]. This study describes the use of a prophylactic multimodal regimen of three to four different antimicrobial agents including a fluoroquinolone, a macrolide, an aminoglycoside, and a benzimidazole in clinically normal rhesus macaques at two different institutions. After the administration of the multimodal treatments, they state that this has created “*Gastrointestinal Pathogen Free (GPF)*” macaques that are more suited for HIV preclinical research. While we believe it is an admirable goal to develop, refine, and characterize new NHP models for HIV research, we are concerned that the authors do not adequately evaluate development of antimicrobial resistance and the associated risks to human and animal health.

## Development of antimicrobial resistance and risk to human health

Antimicrobial resistance (AMR) has been called a ‘silent pandemic’ and is recognized as a global threat to health and development [2]. The effectiveness of antimicrobial agents is diminishing throughout veterinary and human medicine due to the continued rise of antimicrobial resistance in bacterial populations [3–7]. In large part, development of microbial resistance is due to indiscriminate use of antimicrobials exerting selection pressures on microbial species [4].

The authors identify *Shigella* and *Campylobacter* as targets of the proposed multimodal therapy. However, drug resistant *Shigella* and *Campylobacter* are serious threats to human and macaque health. Not only do both *Shigella* and *Campylobacter* have decreasing rates of susceptibility to fluoroquinolones (17% and 28%, respectively) and macrolides (14% and 4%, respectively), but there are reports in both humans and macaques regarding emerging fluoroquinolone-resistant *Shigella* strains [4, 8–10]. A recent study showed that *Shigella flexneri* isolated from macaques contained chromosomal and plasmid-mediated fluoroquinolone resistance [8]. As a result of these resistance patterns, the World Health Organization recently highlighted the need for new antimicrobials to treat fluoroquinolone-resistant *Campylobacter* and *Shigella* [6]. Furthermore, indiscriminate antimicrobial use can also drive resistance in other GI pathogens or in GI commensals which can then pass resistance genes to more pathogenic organisms.

In addition to GI pathogens, antimicrobial use can drive resistance in more distant sites. In humans and animals, fluoroquinolone treatment is a known risk factor for carrying methicillin-resistant *Staphylococcus aureus* (MRSA) [9]. There is increasing emphasis on the need to use the ‘precautionary principle’ when assessing AMR risks, and it is highly plausible that the treatment proposed in Bochart et al. [1] similarly drives MRSA selection in macaques. The multi-antimicrobial regimen described was implemented many years ago, albeit without the use of azithromycin, at the National Cancer Institute [11]. Several years after implementation, 28% (80/288) of animals were found to have MRSA in their nares [12]. A program of antimicrobial stewardship was initiated, including elimination of the multi-antimicrobial regimen described in Bochart in a large proportion of the animals. Three years later, MRSA prevalence was reduced to 9.1%, despite the colony being generally older and animals undergoing more invasive procedures. Antimicrobials should be targeted to animals with clinical indications, in consultation with veterinary staff, and based, as often as possible, on culture and sensitivity results, rather than being widely administered within an NHP colony [13].

Any event that promotes the development of multi-drug resistant bacteria within NHPs puts this scarce and valuable research resource at risk. If AMR bacteria become more common in NHPs, potential spillover events may occur to humans leading to both serious public health consequences and erosion of public trust in our research endeavors. Spillover events have previously been demonstrated with documented transmission of *Shigella* as well as MRSA from NHPs to human staff [14, 15]. Furthermore, in a captive chimpanzee colony, a considerably high prevalence of MRSA (~69%) likely indicates that a human- to- animal transmission event occurred in the past, and that there is potential for further animal-to-human transmission to occur [16]. Additionally, multidrug resistant infections can impact NHP health and welfare, drive a need for treatment with treatment with higher tier antimicrobials (e.g., carbapenems, glycopeptides), and jeopardize research when infected animals must be removed. The COVID-19 pandemic has profoundly impacted NHP availability, thus, all measures to preserve existing NHP colony well-being must be prioritized.

## Concerns regarding conclusions that antimicrobial resistance did not develop and successful creation of GPF status

The authors have suggested that they did not identify antimicrobial resistance associated with this pre-treatment regimen. However, they have not provided sufficient evidence to support this claim. The authors did not monitor any bacterial resistance patterns before or after the treatment or report antimicrobial susceptibility of common bacteria identified within the colony. Assessment of enrichment for *Enterobacteriaceae*, because they are linked to multi-drug resistance, does not factor in selective pressure for resistance genes in other bacterial taxa, including but not limited to *Pseudomonas* spp. (Pseudomonadaceae), *Staphylococcus* spp. (Staphylococcaceae), and *Enterococcus* spp. (Enterococcaceae), all of which have had resistant isolates identified in research macaques [15, 17–19]. As a phenomenon that develops through mutations and gene transfer and where the complication (AMR) accumulates over time, short term, small size studies are inadequate for assessment of AMR emergence.

The small size of the cohort used at ONPRC (n = 16) was designed as a before-and-after study with no control group. The authors acknowledge that the vast majority of animals screened did not meet their selection criteria and that the animals presented were healthier than the average macaque at the start of the study. The resulting selection bias ignores the potential to examine the increased potential for antimicrobial resistance development in animals that are less healthy, or that had recent previous antimicrobial exposure.

In the second portion of the study, the authors state that *Campylobacter* spp. were below the limit of detection by 16S sequencing, and that neither *Campylobacter* nor *Shigella* were detected on rectal culture. *Shigella* is indistinguishable from *E*. *coli* on 16S V4 sequencing and no *Shigella-*specific molecular identification methods were utilized. Given that only 1/16 animals were *Shigella-*positive on culture prior to treatment and that *Shigella* is shed intermittently and difficult to culture in healthy macaques [20], it is not possible to claim eradication, especially given that these animals were only followed for a little over 6 months.

## Concerns about translatability and research reproducibility

The argument made by Bochart et al. is that by reducing the variability of gut inflammation one enhances standardization of the model and thus fewer animals are needed for studies. However, we believe that the authors’ conclusions may fall victim to the standardization fallacy, whereby reducing biological variability actually reduces the scientific advances made and may then increase animal usage [21]. If NHP colonies for HIV studies used the clean-up regimen proposed by Bochart et al., broadly, the value of the heterogeneous NHP model could be lost with unknown consequences leading to less translatable findings or smaller incremental advances requiring more studies and more animals. Severe restrictions of this approach should be applied and, if implemented at all, this approach should be implemented as narrowly as possible. Additionally, IACUCs and reviewers should actively question the scientific need for such endeavors and consider the preservation of systemic heterogenization of NHP subjects within study groups. Research translatability may be best achieved by embracing the biological diversity as exists within human populations.

Finally, if this approach is used, it must be reported in the literature in the methods section of papers to promote research reproducibility. Given the significant impact that pre-study antimicrobials can have on the microbiome of asymptomatic macaques [22] and the observations associated with mucosal inflammation [1], reporting such treatments must be a requirement in the materials and methods of all publications, regardless of the aims of the research.

## Conclusion

While reducing and refining the use of primates in research are goals shared by reasonable persons, the particular method of doing so proposed by Bochart et al. risks both NHP and human health. The practice of “cleaning-up” clinically healthy NHPs with multiple antimicrobials is dangerous, cuts against the basic principles of antimicrobial stewardship [13], and may lead to reduced research translatability, paradoxically increasing the use of animals. We believe the concerns raised by this letter should be critically considered by all readers before implementing the practices described.
